# Extracellular Electron Transfer via Outer Membrane Cytochromes in a Methanotrophic Bacterium *Methylococcus capsulatus* (Bath)

**DOI:** 10.3389/fmicb.2018.02905

**Published:** 2018-11-29

**Authors:** Kenya Tanaka, Sho Yokoe, Kensuke Igarashi, Motoko Takashino, Masahito Ishikawa, Katsutoshi Hori, Shuji Nakanishi, Souichiro Kato

**Affiliations:** ^1^Graduate School of Engineering Science, Osaka University, Toyonaka, Japan; ^2^Department of Biomolecular Engineering, Graduate School of Engineering, Nagoya University, Nagoya, Japan; ^3^Bioproduction Research Institute, National Institute of Advanced Industrial Science and Technology (AIST), Sapporo, Japan; ^4^Research Center for Solar Energy Chemistry, Osaka University, Toyonaka, Japan

**Keywords:** extracellular electron transfer, methanotroph, *Methylococcus capsulatus* (Bath), *c*-type cytochrome, current production, iron reduction

## Abstract

Electron exchange reactions between microbial cells and solid materials, referred to as extracellular electron transfer (EET), have attracted attention in the fields of microbial physiology, microbial ecology, and biotechnology. Studies of model species of iron-reducing, or equivalently, current-generating bacteria such as *Geobacter* spp. and *Shewanella* spp. have revealed that redox-active proteins, especially outer membrane *c*-type cytochromes (OMCs), play a pivotal role in the EET process. Recent (meta)genomic analyses have revealed that diverse microorganisms that have not been demonstrated to have EET ability also harbor OMC-like proteins, indicating that EET via OMCs could be more widely preserved in microorganisms than originally thought. A methanotrophic bacterium *Methylococcus capsulatus* (Bath) was reported to harbor multiple OMC genes whose expression is elevated by Cu starvation. However, the physiological role of these genes is unknown. Therefore, in this study, we explored whether *M. capsulatus* (Bath) displays EET abilities via OMCs. In electrochemical analysis, *M. capsulatus* (Bath) generated anodic current only when electron donors such as formate were available, and could reduce insoluble iron oxides in the presence of electron donor compounds. Furthermore, the current-generating and iron-reducing activities of *M. capsulatus* (Bath) cells that were cultured in a Cu-deficient medium, which promotes high levels of OMC expression, were higher than those cultured in a Cu-supplemented medium. Anodic current production by the Cu-deficient cells was significantly suppressed by disruption of MCA0421, a highly expressed OMC gene, and by treatment with carbon monoxide (CO) gas (an inhibitor of *c*-type cytochromes). Our results provide evidence of EET in *M. capsulatus* (Bath) and demonstrate the pivotal role of OMCs in this process. This study raises the possibility that EET to solid compounds is a novel survival strategy of methanotrophic bacteria.

## Introduction

Extracellular electron transfer (EET) is the process by which some microorganisms exchange intracellular electrons with an extracellular electron donor/acceptor, including naturally occurring metal compounds and artificial electrodes, across the cell membrane (Lovley, 2008; Kato, 2015). Microorganisms harboring EET abilities have received considerable attention as the biocatalysts of bioelectrochemical systems, such as microbial fuel cells and microbial electrosynthesis (Lovley and Nevin, 2013; Igarashi and Kato, 2017; Sasaki et al., 2018). Intensive study of the molecular mechanisms of EET in model microorganisms such as *Geobacter sulfurreducens*, *Shewanella oneidensis*, and *Acidithiobacillus ferrooxidans*, has revealed that redox-active proteins, especially outer membrane *c*-type cytochromes (OMCs), play an important role in EET (Weber et al., 2006; Castelle et al., 2008; Shi et al., 2016). Recent studies have demonstrated that phylogenetically and physiologically diverse microorganisms, including sulfate-reducing bacteria, acetogenic bacteria, filamentous Chloroflexi bacteria, and methanogenic archaea, exhibit EET abilities (Bond and Lovley, 2002; Nevin et al., 2010; Yamada et al., 2014; Kato et al., 2015; Deng et al., 2018; Kawaichi et al., 2018). Furthermore, metagenomics analysis of environmental samples revealed that various uncultured microorganisms, including anaerobic methane-oxidizing archaea, harbor OMC-like genes (McGlynn et al., 2015). Considering that there has been no report that OMCs are used for metabolic reactions other than EET, we can assume that EET via OMCs could be more widely conserved in microorganisms than originally thought.

Aerobic methane-oxidizing bacteria (methanotrophs) utilize methane as their sole carbon and energy source, and play a significant role in global climate by mitigating emissions of methane into the atmosphere. Methanotrophs have also received considerable attention as biocatalysts for conversion of methane into valuable chemicals (Strong et al., 2016; Cantera et al., 2018; Hwang et al., 2018). *Methylococcus capsulatus* (Bath) is a model methanotroph whose physiology, biochemistry, and genetics have been extensively investigated (Kelly et al., 2005; Hakemian and Rosenzweig, 2007). Availability of Cu markedly affects the gene expression and physiology of *M. capsulatus* (Bath) by a process called the “copper switch” (Semrau et al., 2018). *M. capsulatus* (Bath) uses two different methane monooxygenases (MMOs) according to the availability of Cu: a Cu-containing particulate enzyme (pMMO) is highly expressed in the presence of Cu, whereas the soluble counterpart (sMMO) predominantly functions in the absence of Cu (Semrau et al., 2018). In addition to the MMO enzymes, some OMC-like genes in *M. capsulatus* (Bath) are highly expressed in the absence of Cu (Karlsen et al., 2005, 2008). Furthermore, some of the OMC genes form a gene cluster with a gene homologous to the β-barrel outer membrane protein (*mtrB*) of *S. oneidensis*, which has been observed to complex with OMCs in the outer membrane (Larsen and Karlsen, 2016). Based on these findings, we considered it likely that *M. capsulatus* (Bath) possesses EET capability via OMCs; however, no experimental evidence has been provided for the existence of EET capability in methanotrophs, and the physiological functions of their OMCs have not been clarified.

In this study, the EET ability of *M. capsulatus* (Bath) was verified by assessing current-producing and Fe(III)-reducing activities of cells cultured in the presence or absence of Cu. The contribution of OMCs to the EET ability was also verified by investigating the effects of deletion of an OMC gene and supplementation of carbon monoxide (CO) (an inhibitor of cytochromes) on current-producing activities.

## Materials and Methods

### Bacterial Strains and Culture Conditions

*Escherichia coli* strains were grown in Luria-Bertani medium at 37 °C with shaking. Gentamycin (10 μg/mL) and 600 μM 2,6-diaminopimelic acid (DAP) were added into the medium when required. *M. capsulatus* (Bath) and its derivative mutant were cultured in test tubes (26 mL capacity) filled with 5 mL of inorganic basal medium [5 mM NaNO_3_, 2 mM KH_2_PO_4_, 1 mM MgCl_2_, 0.1 mM Na_2_SO_4_, and 20 mM HEPES (4-{2-hydroxyethyl}-1-piperazineethanesulfonic acid), pH 7.0], and 10 mL/L each of trace element solution (-CuCl_2_) and vitamin solution (Kato et al., 2016). The test tubes were sealed with butyl rubber stoppers and aluminum seals before autoclave sterilization. Depending on the experiment, 20 μM CuCl_2_ was added from sterilized stock solution after autoclaving. Cultures were supplemented with methane (2 mL/tube) as the carbon and energy source and incubated at 37°C with shaking. All culture experiments were conducted in triplicate and statistically analyzed by the Student’s *t*-test.

### Quantitative Reverse Transcription-PCR Analysis

Total RNA was isolated from *M. capsulatus* (Bath) cells by using a bead-beating method as described previously (Kato et al., 2014). The PCR primers used for quantitative reverse transcription (qRT)-PCR were designed with Primer3 software (Untergasser et al., 2012) and are listed in Supplementary Table S1. Quantitative expression analysis based on one-step real-time RT-PCR was performed using RNA-direct SYBR Green Realtime PCR Master Mix (Toyobo, Osaka, Japan) and the Mx3000P System (Stratagene, San Diego, CA, United States) as described previously (Kato et al., 2014). The gene for outer membrane protein B (*mopB*), a housekeeping gene of *M. capsulatus* (Bath) (Karlsen et al., 2005), was used to normalize expression values.

### Fe(III) Reduction Assay

*Methylococcus capsulatus* (Bath) was cultured until the early stationary phase in the presence or absence of Cu as described above. Cells were collected by centrifugation, washed twice with the Cu-free basal medium, and suspended in the same medium such that the optical density at 540 nm was 0.5. Ferrihydrite was prepared as described previously (Lovley and Phillips, 1986), and added to the cell suspension to give a final concentration of 10 mM Fe. Sodium formate (final concentration, 10 mM) was added from a sterilized stock solution. The cell suspension was incubated at 37°C under N_2_ atmosphere with shaking. The concentration of Fe(II) was determined by the ferrozine method as described previously (Lovley and Phillips, 1987).

### Electrochemical Analysis

All electrochemical measurements were carried out at 37°C in a single-chamber electrochemical cell with an indium-tin oxide electrode (3 cm^2^), an Ag/AgCl (saturated KCl) electrode, and a platinum wire as the working, reference, and counter electrode, respectively. After 3 mL of the Cu-free basal medium containing 10 mM sodium formate was introduced into the electrochemical cell, the working electrode potential was set at +0.4 V against the reference electrode by using a potentiostat (HA-1510, Hokuto Denko, Tokyo, Japan) and the current was recorded by a data logger [NR-1000 Data Acquisition System (Keyence, Osaka, Japan)]. The *M. capsulatus* (Bath) cells were cultured in the basal medium in the presence or absence of Cu, collected by centrifugation, suspended in 1 mL Cu-free basal medium with a final optical density at 540 nm of 2.0, and then injected into the electrochemical cell.

### Construction of a Deletion Mutant

A deletion mutant of the OMC gene MCA0421 was constructed by the double crossover method by using mutated *pheS* as the counter-selectable marker as previously described (Ishikawa et al., 2018). Primers used for construction of the mutant strain are listed in Supplementary Table S2. Briefly, 1-kb regions upstream and downstream of MCA0421 were amplified by PCR using primer sets MCA0421_upstF/MCA0421_upstR and MCA0421_dwstF/MCA0421_dwstR, respectively. These fragments were cloned into the *Bam*HI sites of pJQY3 by using the NEBuilder Hifi DNA assembly system (New England Biolabs, Ipswich, MA, United States), generating pJQY2. *M. capsulatus* (Bath) was mated with *E. coli* WM6026 (Blodgett et al., 2007) harboring pJQY2 on a nitrate mineral salts (NMS) agar plate (Whittenbury et al., 1970) supplied with 600 μM DAP under the air-methane gas mixture (80:20) at 37°C for 24 h. The cells were collected in 500 μL of NMS medium, plated on an NMS agar plate containing gentamicin (10 μg/mL), and incubated under the air-methane gas mixture (80:20) at 37°C until colonies were generated. The resultant candidate single crossover mutant was grown in NMS medium without gentamycin for 3–4 days and its dilutions were placed on an NMS agar plate containing 10 mM *p*-chloro-phenylalanine. After incubation under the air-methane gas mixture (80:20) at 37°C, the colonies were screened for deletion of MCA0421 by PCR using primers MCA0421_SeqF and MCA0421_SeqR. The DNA amplicon was sequenced to confirm successful deletion of MCA0421.

## Results and Discussion

### Effects of Cu Availability on OMC Expression

**FIGURE 1:**
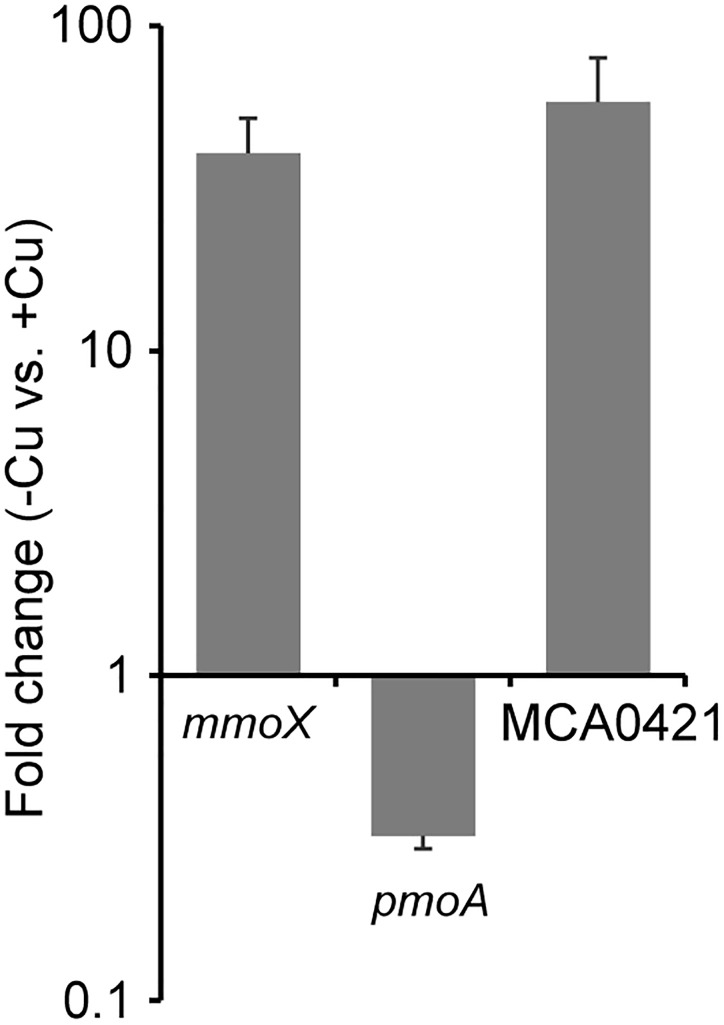
Effects of Cu availability on the expression of genes for soluble methane monooxygenase (sMMO) (*mmoX*), particulate methane monooxygenase (pMMO) (*pmoA*), and an outer membrane *c*-type cytochrome (OMC) (MCA0421). *Methylococcus capsulatus* (Bath) was cultured in the presence and absence of Cu (+Cu and –Cu, respectively), and subjected to quantitative reverse transcription (qRT)-PCR analysis. The expression values of each gene were normalized with that of a house-keeping gene (membrane protein B). The vertical axis represents the expression fold changes of –Cu vs. +Cu. Data are presented as means of triplicate experiments, and error bars represent standard deviation.

To verify the EET ability of *M. capsulatus* (Bath), culture conditions that induce different OMC expression levels were examined. Cells were cultured in inorganic basal medium in the presence or absence of 20 μM CuCl_2_ until the late logarithmic phase. The expression level of the MCA0421 gene, which encodes a highly expressed OMC (Karlsen et al., 2005, 2008), was measured by qRT-PCR (Figure 1). Genes for two types of MMOs, *mmoX* and *pmoA*, whose expressions are up- and down-regulated by Cu starvation, respectively (Semrau et al., 2018), were analyzed as controls. Consistent with the previous studies, *mmoX* was up-regulated (41 fold, *p* < 0.05) in the Cu-deficient condition, whereas *pmoA* was down-regulated (0.32 fold, *p* < 0.05). MCA0421 gene expression was significantly higher (58 fold, *p* < 0.05) in the absence of Cu than in the presence of Cu. Therefore, *M. capsulatus* (Bath) cultured in Cu-deficient and -supplemented conditions were utilized in the following experiments as models of cells highly or poorly expressing OMCs, respectively.

### Ferrihydrite Reduction by *M. capsulatus* (Bath)

**FIGURE 2:**
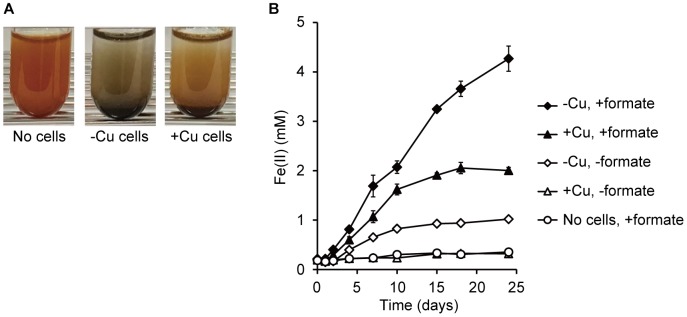
Reduction of ferrihydrite by *M. capsulatus* (Bath). **(A)** Changes in color of ferrihydrite particles after 15-d incubation in the absence (“No cells”) or presence of *M. capsulatus* (Bath) cells. Cells were prepared under Cu-deficient (–Cu) or Cu-supplemented (+Cu) conditions. **(B)** Production of Fe(II) via reduction of ferrihydrite by the –Cu and +Cu cells of *M. capsulatus* (Bath) in the presence or absence of formate. Data are presented as means of triplicate experiments, and error bars represent standard deviation.

To determine the EET capabilities of *M. capsulatus* (Bath), the reduction of insoluble iron oxides (ferrihydrite) by *M. capsulatus* (Bath) cell suspensions was assessed. In the presence of *M. capsulatus* (Bath) cells and an electron donor compound (10 mM formate), ferrihydrite particles that were reddish brown in color turned black (Figure 2A), indicating occurrence of Fe(III) reduction. *M. capsulatus* (Bath) cells cultured in Cu-deficient medium (referred to as -Cu cells, which highly expressed OMCs) produced Fe(II) via ferrihydrite reduction in the presence of formate (Figure 2B). Ferrihydrite reduction by the -Cu cells also occurred in the absence of formate, whereas the reduction activities were much lower. Organic compounds accumulated in the cells during pre-culture may serve as electron donors for Fe(III) reduction. *M. capsulatus* (Bath) cells cultured in Cu-supplemented medium (referred to as +Cu cells, which poorly expressed OMCs) also reduced ferrihydrite, but the activity was much lower than that by the -Cu cells. Fe(III) reduction by *M. capsulatus* (Bath) was also observed when methanol was used as the electron donor instead of formate (Supplementary Figure S1). The same trend was observed for the effect of Cu deficient pre-culture, while the Fe(III) reduction activity with methanol was lower than that with formate. These results indicate that *M. capsulatus* (Bath) has EET capability and that their OMCs are involved in the EET process. We also assessed the growth of *M. capsulatus* (Bath) under the Fe(III)-reducing conditions; however, no growth was observed in the absence of oxygen even when methane or formate was added as an electron donor (data not shown), indicating that EET reactions are not a sufficient means of energy acquisition to support growth of *M. capsulatus* (Bath).

### Anodic Current Production by *M. capsulatus* (Bath)

**FIGURE 3:**
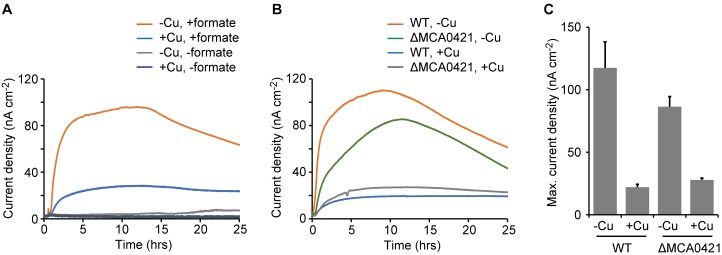
Anodic current production by wild-type (WT) and OMC-deletion mutant (ΔMCA0421) cells of *M. capsulatus* (Bath). **(A)** Anodic current production by WT cells in the presence or absence of formate. **(B)** Anodic current production by WT and ΔMCA0421 cells. **(A,B)** *M. capsulatus* (Bath) cells were pre-cultured under Cu-deficient (–Cu) or Cu-supplemented (+Cu) conditions. Data plots are representative of at least three independent experiments. **(C)** Comparison of the maximum current densities calculated from the data of A and B. Data are presented as means of triplicate experiments, and error bars represent standard deviation.

To quantitatively evaluate the EET activities of *M. capsulatus* (Bath), anodic current production (i.e., electron transfer from microbial cells to an anode) was measured in an electrochemical cell (See Materials and Methods). *M. capsulatus* (Bath) cells prepared from the Cu-deficient culture produced anodic current (maximum current density, 118 ± 20 nA cm^-2^) in the presence of formate (Figures 3A,C). In contrast, the current density was quite low in the control cultures omitting either bacterial cells or formate (<8 nA cm^-2^). *M. capsulatus* (Bath) cells prepared from the Cu-supplemented culture also exhibited anodic current production, but the maximum current density (22 ± 2 nA cm^-2^) was significantly lower than that produced by the -Cu cells (*p* < 0.05, Figure 3C). These results are consistent with the Fe(III) reduction assays, and confirm that *M. capsulatus* (Bath) has EET abilities via OMCs.

### Effects of Deletion of an OMC Gene on Anodic Current Production

To explore the involvement of OMCs in the EET activities, a mutant strain of *M. capsulatus* (Bath) that was deficient in an OMC gene was constructed and its current-generating activity was measured. The MCA0421 gene, which encodes the OMC with the highest expression level under Cu-limited conditions in previous reports (Karlsen et al., 2005, 2008), was selected as the target of disruption. The mutant strain (ΔMCA0421) was constructed by the double crossover method using mutated *pheS* as the counter-selectable marker (see Materials and Methods; Ishikawa et al., 2018). The ΔMCA0421 cells produced anodic currents, and the effects of Cu availability in the pre-cultures were similar to that observed for wild-type (WT) *M. capsulatus* (Bath) (Figure 3B). However, the current produced by ΔMCA0421 cells prepared under the Cu-deficient conditions (maximum current density, 86 ± 8 nA cm^-2^) was significantly smaller than that produced by WT cells (*p* < 0.05, Figure 3C). These results indicate that the major OMC gene, MCA0421, at least partly involves in the EET process of *M. capsulatus* (Bath).

Similar to the results in our study, a decrease but not abolition of EET activities was also observed following deletion of a single OMC gene that is a major contributor to EET activities in other bacterial strains such as *S. oneidensis* and *G. sulfurreducens* (Holmes et al., 2006; Newton et al., 2009). These previous reports propose that multiple OMCs whose expression levels are low in WT are involved in the EET reactions of mutants lacking the major OMCs. Among more than 50 *c*-type cytochrome genes encoded by the genome of *M. capsulatus* (Bath) (Ward et al., 2004), 15 have been confirmed by proteome analysis to be OMCs, in that they are expressed and localized on the cell surface (Larsen and Karlsen, 2016). Among the 15 putative OMCs, 10 have been proved to be up-regulated under Cu-deficient conditions (Larsen and Karlsen, 2016). Therefore, we assume that multiple OMCs, including MCA0421, play pivotal roles in the EET reactions of *M. capsulatus* (Bath). Further investigations are required to clarify the exact roles of multiple OMCs in *M. capsulatus* (Bath).

### Effects of Inhibition of Cytochromes on Anodic Current Production

**FIGURE 4:**
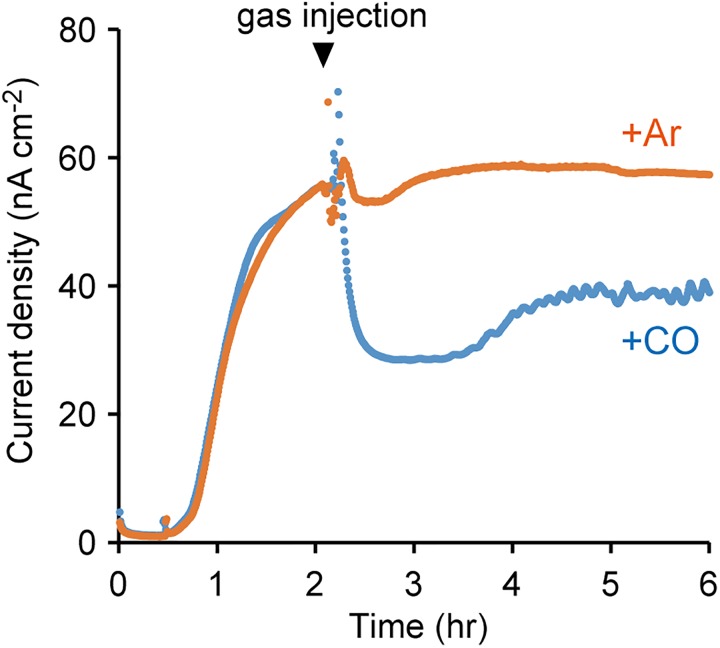
Effects of carbon monoxide (CO) gas on the anodic current production by *M. capsulatus* (Bath). The electrochemical measurements were performed under N_2_ atmosphere, and injection of CO or Ar gas started at the time indicated by the arrowhead. The data plots are representative of at least three independent experiments.

To further elucidate the involvement of OMCs in EET, the effect of an inhibitor of cytochromes on current production by *M. capsulatus* (Bath) was investigated. CO inhibits electron transfer activity of cytochromes by specifically and tightly coordinating to the cytochrome heme groups, and is utilized as the inhibitor of OMC-dependent EET activities in current-generating bacteria (Shibanuma et al., 2011; Ishii et al., 2015). Upon the treatment of *M. capsulatus* (Bath) cells with CO, the anodic current sharply decreased (Figure 4). In contrast, the current production was not affected when the cells were treated with Ar. These results strongly support the notion that OMCs play pivotal roles in current production by *M. capsulatus* (Bath).

### Physiological and Ecological Implications

This study provides the first experimental evidence of EET capability in methanotrophs. However, the physiological and ecological implications remain unclear. Generally, microorganisms with EET abilities acquire the energy required for growth by using solid compounds such as iron oxides and electrodes as the sole electron acceptor. However, we observed that *M. capsulatus* (Bath) did not grow under Fe(III)-reducing conditions. Furthermore, the EET activity of *M. capsulatus* (Bath) was considerably lower than that of known current-producing and/or iron-reducing bacteria. For example, the current producing activity of *M. capsulatus* (Bath) observed in this study (c.a., 100 nA/cm^2^) is one order of magnitude lower than that of *S. oneidensis* (c.a., several μA/cm^2^) under the same experimental settings (Liu et al., 2010). From these observations, we assume that *M. capsulatus* (Bath) does not employ EET to utilize extracellular solid compounds as the electron acceptor instead of oxygen. Furthermore, *M. capsulatus* (Bath) does not have the advantage of using electron acceptors other than oxygen, because it requires molecular oxygen as a substrate for the MMO reactions.

Hence, we hypothesize that *M. capsulatus* (Bath) employs EET to maintain cellular redox homeostasis. It has been reported that *M. capsulatus* (Bath) compensates for insufficient reducing equivalents by oxidizing molecular hydrogen to supplement methanotrophic growth, but does not grow if molecular hydrogen is the sole electron donor (Hanczár et al., 2002). Similarly, *M. capsulatus* (Bath) might utilize insoluble solid compounds as an additional electron source (or sink) when the cellular redox state becomes oxidative (or reductive). This hypothesis is consistent with our finding that *M. capsulatus* (Bath) cells over-expressed OMCs under Cu-deficient conditions. *M. capsulatus* (Bath) preferentially utilize sMMO instead of pMMO as the methane-oxidizing enzyme under Cu-deficient conditions (Semrau et al., 2018). The methane oxidation reaction requires reducing equivalents for activation of methane (Keltjens et al., 2014). Electrons are supplied to pMMO via reduced quinone or *c*-cytochrome, whereas sMMO requires electron carriers with higher energy such as NAD(P)H. Hence, we propose that reducing power tends to be insufficient under Cu-deficient conditions, and that this induces expression of OMCs to enable the cells to acquire reducing power via EET. Further investigations are required to clarify the physiological and ecological significance of EET in methanotrophic bacteria.

## Author Contributions

SN and SK designed the research. KT and SN conducted the electrochemical experiments. SY, MI, and KH constructed the mutant strain. KI and MT conducted culture experiments. KT, SN, and SK wrote the manuscript with input from all authors.

## Conflict of Interest Statement

The authors declare that the research was conducted in the absence of any commercial or financial relationships that could be construed as a potential conflict of interest.
