# Supplementary material for: Extracellular Electron Transfer via Outer Membrane Cytochromes in a Methanotrophic Bacterium Methylococcus capsulatus (Bath)
Source: Front Microbiol. 2018 Nov 29;9:2905. doi: 10.3389/fmicb.2018.02905 (PMC6281684; doi:10.3389/fmicb.2018.02905)
Supplement: Supplementary file 1 [file Data_Sheet_1.docx]

Supplementary material

Extracellular electron transfer via outer membrane cytochromes in a methanotrophic bacterium *Methylococcus capsulatus* (Bath)

Kenya Tanaka^1^, Sho Yokoe^2^, Kensuke Igarashi^3^, Motoko Takashino^3^, Masahito Ishikawa^2,4^, Katsutoshi Hori^2^, Shuji Nakanishi^1,4^*, and Souichiro Kato^3,4^*

^1^ Graduate School of Engineering Science, Osaka University, Toyonaka, Osaka, Japan

^2^ Department of Biomolecular Engineering, Graduate School of Engineering, Nagoya University, Furo-cho, Chikusa-ku, Nagoya, Japan

^3^ Bioproduction Research Institute, National Institute of Advanced Industrial Science and Technology (AIST), Sapporo, Japan

^4^ Research Center for Solar Energy Chemistry, Osaka University, Toyonaka, Osaka, Japan

* For correspondence:

Dr. Shuji Nakanishi,

E-mail: nakanishi@chem.es.osaka-u.ac.jp

Dr. Souichiro Kato

E-mail: s.katou@aist.go.jp

Fig. S1, Table S1-S2

**Figure S1.** Production of Fe(II) via reduction of ferrihydrite by the −Cu and +Cu cells of *M. capsuatus* (Bath) in the presence or absence of methanol. Data are presented as means of triplicate experiments, and error bars represent standard deviation.

**
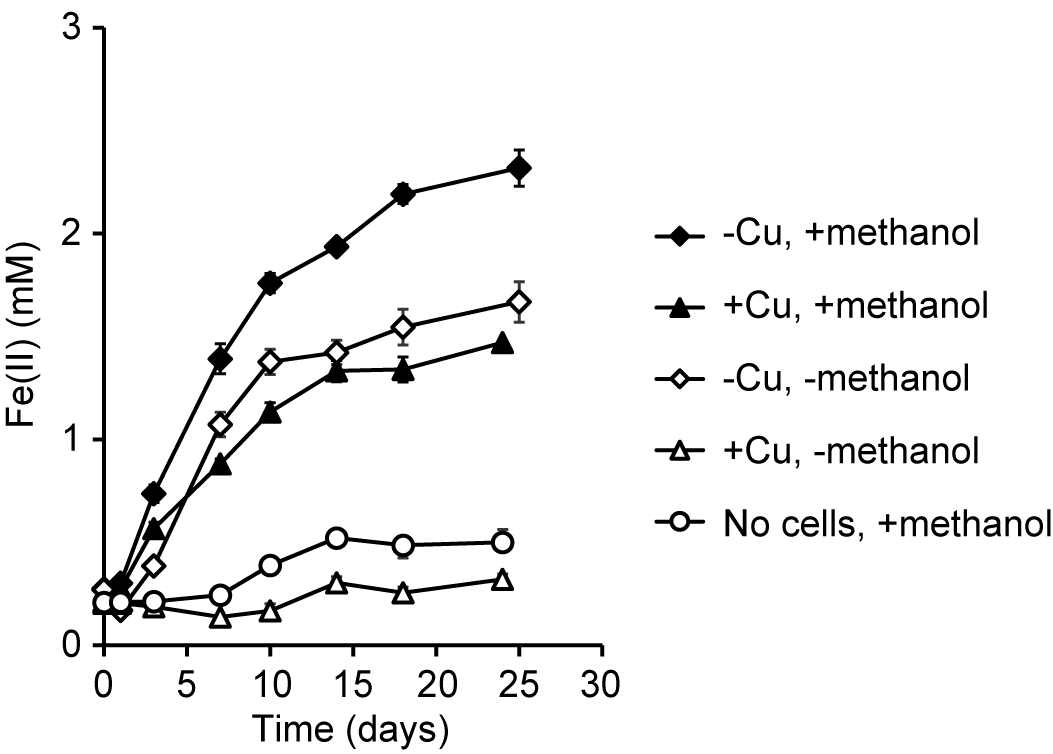
**

**Table S1.** Primers used for qRT-PCR.

| Primer name | Target gene | Sequence (5′ to 3′) |
| --- | --- | --- |
| MmoX_129F | *mmoX* | GAC CAA GTA CGC CAC CAA GT |
| MmoX_332R | *mmoX* | TCC AGG AAG TTC GAA ACC AC |
| PmoA_338F | *pmoA* | ACT TCC CGA TCA ACT TCG TG |
| PmoA_534R | *pmoA* | CAT CAG CAT GCC GTT GTA TT |
| MCA0421_1850F | MCA0421 | TCT ACG ACG CCT ATC CGT TC |
| MCA0421_2041R | MCA0421 | TTG ATG TCG AAA CCC TCC TC |
| MopB_57F | *mopB* | ACA GGC CGA AGA GAC TTT CA |
| MopB_246R | *mopB* | GGT GGT GTT GCC TTC GTA AT |

**Table S2.** Primers used for construction of the mutant strain.

| Primer name | Sequence (5′ to 3′) |
| --- | --- |
| MCA0421_upstF | CGA ATT CCT GCA GCC CGG GGG ATC GGA ACA GGT CGG GAA GAA TG |
| MCA0421_upstR | GGT CTG GAG ATG TCG TAG ATG TCG ATG G |
| MCA0421_dwstF | ATC TAC GAC ATC TCC AGA CCA GCT TCA TC |
| MCA0421_dwstR | CGG CCG CTC TAG AAC TAG TGG ATC CGG GAT AAG GAT TGG CGT TC |
| MCA0421_SeqF | GTG AAC AGG ATG AAC AGC AGG ATG CCG ACG |
| MCA0421_SeqR | GTT GAG GGT GTT GTA AGG AAT GAG CGG ATT G |
